# An analysis of global youth tobacco survey for developing a comprehensive national smoking policy in Timor-Leste

**DOI:** 10.1186/s12889-016-2742-5

**Published:** 2016-01-22

**Authors:** Decio Ribeiro Sarmento, Degninou Yehadji

**Affiliations:** 1 Fulbright SERN Timor-Leste Awardee for School of Public Health, Georgia State University, Atlanta, 30303 Georgia; 2 Fulbright Awardee Togo for School of Public Health, Georgia State University, Atlanta, 30303 Georgia

**Keywords:** Current smoking prevalence, Tobacco use, Smoking in Timor-Leste, Smoking among minors, Smoking policy, Tobacco control program

## Abstract

**Background:**

Smoking is a global public health concern. Timor-Leste is facing a rapidly growing epidemic of tobacco use. The trend of smoking in Timor-Leste seems to be increasing and the magnitude of the problem affects people who smoke before reaching adulthood. One of the factors implicated in the continuously rising trend of smoking among young people in Timor-Leste is clearly due to unavailability of restrictive laws and regulations. Therefore, our study sought to analyze available dataset from the Global Youth Tobacco Survey (GYTS) for developing a comprehensive national smoking policy in order to lower smoking risks among young people in Timor-Leste.

**Methods:**

We conducted a secondary analysis of the 2009 GYTS in Timor-Leste. The 2009 GYTS assessed 1657 in-school students aged 13–15 years for current smoking prevalence and determinants of tobacco use. We used IBM SPSS version 21 software to analyze the data. Frequency analyses were computed to identify demographic characteristics of study participants. Bivariate logistic regression analysis was performed to examine the association between each demographic characteristic as well as each independent variable and the outcome of being current smokers.

**Results:**

Out of 1657 in-school students, 51 % were of ages less than 15; 53 % were girls; and 45 % were in grade 2. Prevalence of current cigarette smoking was found to be 51 %. The prevalence of current smoking among in-school students increased with ages (from 46 % in less than 15 to 57 % in 15 plus). Boys were more likely to be smokers than girls (59 % versus 28 %). Significant factors positively associated with current smoking included parental smoking; closed-peer smoking; number of days people smoked in the house; having family discussion about harmful effects of smoking; being smoking in areas such as school, public places and home; and having seen cigarette advertisements on billboard.

**Conclusion:**

Timor-Leste has higher prevalence of cigarette smoking among minors, especially among boys. Our analysis provides evidence-based information for developing comprehensive tobacco control programs - both education and policy interventions to reduce smoking rate among young people in Timor-Leste.

**Electronic supplementary material:**

The online version of this article (doi:10.1186/s12889-016-2742-5) contains supplementary material, which is available to authorized users.

## Background

The use of tobacco by adolescents remains a major public health concern worldwide. There are 1.2 billion people who smoke globally, of whom more than 50 % are young people [1]. This source also reports that in the Southeast Asia and Pacific Region, including Timor-Leste, there are about 600 million tobacco users, of whom 90 % begin under the age of 18. Current estimate by the World Health Organization (WHO) illustrates that tobacco use has caused 5.4 million deaths in 2004 and 100 million deaths over the course of the 21st century [2]. Tobacco smoking has been found to have impact on both economic and health.

Mounting evidence suggests that the economic impact of tobacco smoking has been obvious, especially in low and middle-income countries where many of the poorest smokers tend to spend significant amounts of their income on tobacco instead of basic human needs such as food, shelter, healthcare and education. In Egypt, lower income households are likely to spend more than 10 % of household expenditures on cigarettes or other forms of tobacco [3]. A study carried out in Minhang district of China found that smokers spent 60 % of their personal income and 17 % of household income was likely to be spent on cigarettes [4]. This source also reports that smoker’s family in the Philippines is likely to spend 20 % of the household income on tobacco.

In addition, causal relationships have been established between tobacco use and some adverse health outcomes [5]. Smoking can increase the probability of developing chronic diseases such as lung cancer, cardiovascular diseases, chronic airways diseases, premature births and many other conditions [6]. Recent epidemiologic and experimental findings show that cigarette smoking can be highly associated with the risk of getting lung cancer [7] and cardiovascular diseases [8]. Smoking has also been found to increase the risk of developing *Mycobacterium tuberculosis* infection, which accounts for high mortality in developing countries [9]. Therefore, smoking has become one of the major preventable indicators for mortality and morbidity. Since smoking can harm people’s health, it has become an emerging public health concern in both the developed and developing world including in Timor-Leste.

Smoking in Timor-Leste is a serious problem and justifies more effort to stop it, or better, to prevent young people from smoking initiation. Despite lack of national statistical data, the trend of smoking in Timor-Leste seems to be increasing and the magnitude of the problem affects people who smoke before reaching adulthood. One of the factors implicated in the continuously rising trend of smoking among adolescents in Timor-Leste is clearly due to unavailability of restrictive laws and regulations [10]. Cigarettes are available in small shops and street vendors in Timor-Leste; at the same time regulation on tobacco use has not yet particularly focused on controlling the tobacco access among young people.

Timor-Leste has signed up for the WHO Framework Convention on Tobacco Control (FCTC) since 2004, thus committing itself to develop, implement and evaluate effective tobacco control programs. The intervention will be more appropriate if it is comprehensive and includes policy development to provide regulation to limit access on tobacco use among younger age (minors). Although a study was carried out to investigate prevalence and correlates of current cigarette smoking among young people in Timor-Leste in 2006 [11], surprisingly, little was known and discussed about how policy could play a tremendous role in tobacco control program. Therefore, the purpose of our research was to address the question of whether the development of a comprehensive tobacco control program including policy intervention can lower smoking risks among young people in Timor-Leste. By analyzing available dataset from the 2009 GYTS, we hypothesize that developing a comprehensive smoking policy is tremendously required to address the issue of high smoking prevalence among minors in Timor-Leste. Evidence-based information and analysis can support programs to reduce the number of deaths and disease burden associated with smoking risk factors. The significance of our analysis would enable Timor-Leste’s government to develop its first national smoking policy to be existed in the country after 15 years of independence and to inform decision-making in order to allocate more resources towards tobacco control program. The findings of this analysis would also serve as baseline information for the Ministry of Health (MoH) of Timor-Leste to effectively and efficiently plan, implement and evaluate tobacco control programs.

## Methods

### Data source

We conducted a secondary analysis of the GYTS. Timor-Leste carried out the GYTS in 2006 and 2009 and data from these surveys could be used as baseline for evaluation of the tobacco control programs implemented by the MoH, and for development of national smoking policy by the policy makers of the Timor-Leste government.

The GYTS was developed by the WHO and the Centers for Disease Control and Prevention (CDC) to track tobacco use among young people and enhance the capacity of countries to design, implement, and evaluate tobacco control and prevention programs. The GYTS is a school-based surveillance system designed to allow countries throughout the world to track youth tobacco use in a common, standardized format. Standard methodology guided the sampling and selection procedures, preparation of questionnaires, and ensured consistency of data collection, management, and analysis. The GYTS used a two-stage cluster sample design that produced representative samples of students in grades 7 to 9, which were associated with ages 13–15. The survey sought to collect information in seven major areas: knowledge and attitudes toward cigarette smoking, prevalence of all tobacco use, the influence of media and advertising on the use of cigarettes, accessibility of cigarettes, tobacco-related curriculum in schools, exposure to secondhand smoke, and smoking cessation.

### Study design and participants

The GYTS was a cross sectional analysis, using a survey. The survey included a self-administered questionnaire with 56 core questions. The questionnaire has been annexed in the final report of the survey, which can be accessed online http://dhsprogram.com/pubs/pdf/fr235/fr235.pdf. Through a multi-stage sampling design, schools were selected in the first stage proportional to their enrollment size; then, the second stage, classrooms within each school were randomly selected, with all students in the class eligible to participate. Student participation was voluntary and anonymous using self-administered data-collection procedures.

The Timor-Leste GYTS included data on prevalence of cigarette and other tobacco use as well as information on five determinants of tobacco use: access/availability and price, exposure to second-hand smoke (SHS), cessation, media and advertising, and school curriculum. In 2009, Timor-Leste conducted the GYTS, which was a school-based survey of students in grades 1, 2, and 3 (equivalent to grades 7, 8 and 9 in other settings). A two-stage cluster sample design was used to produce representative data for Timor-Leste. At the first stage, schools were selected with probability proportional to enrollment size. At the second stage, classes were randomly selected and all students in selected classes were eligible to participate. The 2009 GYTS Timor-Leste data had 96 % school response rate with 100 % class response rate and 80 % student response rate, so the total response rate was 77 %. A total of 1657 students aged 13–15 participated in the 2009 GYTS.

### Selection of variables and statistical methods

The outcome variable was the current cigarette smoking status, which was defined as having smoked a cigarette, even in a single puff, in the last 30 days preceding the survey. The independent variables included the demographic variables such as age, gender and education level by grades and some other significant variables such as parental and close friends smoking, areas of smoking, family discussion about harmful effects of smoking and exposure to tobacco related advertisements. These selected predictors, found to be proximate causes of high smoking rate among adolescents [11], are components that Timor-Leste should include in a comprehensive tobacco control program and can consider for developing an evidence-based national smoking policy.

Our data analysis was performed using IBM SPSS version 21 software. Descriptive analyses were computed to identify the demographic characteristics of study participants in the 2009 Timor-Leste GYTS. Bivariate logistic regression analysis was performed to examine the association between each demographic variable as well as each independent variable and the outcome of being current smoker. The level of significance was α = .05. This analysis did not analyze correctly as survey sample; therefore, a sampling weight was not applied.

### Ethics review and written consent

The 2009 GYTS received ethics approval from the Timor-Leste Ministry of Health. Written informed consent for participation in the study was obtained from participants.

## Results

### Descriptive analysis

The demographic characteristics of study participants are displayed in Table 1. A total of 1657 in-school students participated in the Timor-Leste GYTS in 2009. Overall, 51 % of the participants were of ages less than 15, 53 % were girls, and 45 % were in grade 2. Prevalence of current cigarette smoking was found to be 51 %.

**Table 1:** Demographic characteristics of study participants in the Timor-Leste GYTS 2009

Characteristics	Total
	N (%)
Age (years)	
<15	817 (51.0 %)
15 +	798 (49.0 %)
Gender	
Boys	752 (47.0 %)
Girls	849 (53.0 %)
Education level (grades)	
Grade 1	445 (27.0 %)
Grade 2	732 (45.0 %)
Grade 3	439 (27.0 %)
Currently smoking	
Yes	667 (51.0 %)
No	649 (49.0 %)

### Bivariate analysis

The results of bivariate logistic regression analysis of the associations between each demographic variable and current smoking status are shown in Table 2. An estimate for overall smoking rate amongst young people was 51 %. The prevalence of current smoking status was higher among in-school students with more than 15 years of age (*p*-value < .05). The prevalence of current smoking status was higher in boys than girls (*p*-value < .05). Students in grade 1 had higher prevalence of current smoking status (53 %), but the association was not statistically significant (*p*-value > .05).

**Table 2:** Association between each demographic variable and current smoking status among in-school students in Timor-Leste (an estimate of smoking rate is 51.0 %)

Characteristics	Current smoking status		*P*-value
	Yes	No	
	N (%)	N (%)	
Age			
<15	299 (46.0 %)	365 (54.0 %)	<0.05
15+	351 (57.0 %)	270 (43.0 %)	
Gender			
Boys	380 (59.0 %)	177 (41.0 %)	<0.05
Girls	236 (28.0 %)	457 (72.0 %)	
Education level (grades)			
Grade 1	175 (53.0 %)	156 (47.0 %)	>0.05
Grade 2	292 (49.0 %)	304 (51.0 %)	
Grade 3	178 (50.0 %)	180 (50.0 %)	

The results of bivariate logistic regression analysis of the association between each independent variable of interest and the outcome of current smoking status are displayed in Table 3. The prevalence of current smoking status was higher in students who had parents and close friends smoke (*p*-value < .05). The prevalence of current smoking status among in-school students was higher when they had seen people smoking in the house for more than 1 day (*p*-value < .05). Furthermore, the prevalence of current smoking status was higher among students who had family discussion about harmful effects of smoking (*p*-value < .05). In terms of areas of smoking, the primary sites of smoking were schools (97 %), the secondary sites were public places (81 %) and the lowest prevalence was observed among participants smoking at home and friend’s houses (59 %). Participants who saw cigarette advertisements on billboard also had higher prevalence of smoking (60 %).

**Table 3:** Association between each independent variable and current smoking status among in-school students in Timor-Leste

Independent variables	Current smoking status		*P*-value
	Yes	No	
	N (%)	N (%)	
Parents smoked			
Yes	453 (69.0 %)	392 (31.0 %)	<0.05
No	144 (22.0 %)	208 (78.0 %)	
Don’t Know	56 (91.0 %)	41 (9.0 %)	
Close friends smoked			
Yes	507 (76.0 %)	286 (24.0 %)	<0.05
No	157 (44.0 %)	360 (56.0 %)	
Number of days the participant has seen people smoking in the house (last 7 days)			
0	203 (31.0 %)	335 (69.0 %)	<0.05
More than 1	455 (52.0 %)	309 (48.0 %)	
Family discussion about harmful effects of smoking			
Yes	382 (50.0 %)	284 (50.0 %)	<0.05
No	261 (41.0 %)	359 (59.0 %)	
Areas of smoking			
Home and friend’s house	271 (59.0 %)	28 (41.0 %)	<0.05
School	19 (97.0 %)	4 (3.0 %)	
Public spaces (parks, street corners,	128 (81.0 %)	5 (19.0 %)	
shopping centers,			
social events)			
Never smoked	246 (37.0 %)	610 (63.0 %)	
Cigarette advertisements seen on billboard			
Yes	432 (60.0 %)	246 (40.0 %)	<0.05
No	211 (54.0 %)	154 (46.0 %)	

## Discussion

### Prevalence of current smoking status among minors (ages 13–15) in Timor-Leste

Our main findings demonstrate that an overall prevalence of current cigarette smoking status among minors in Timor-Leste is 51 % with rate among boys of 59 % and girls of 28 % respectively. This trend has increased quite dramatically since the previous GYTS conducted in 2006, which was about 32 % (with boys and girls reported 50 and 17 % of being current smokers respectively at that period) [12]. The figure shows that the prevalence of current smokers among minors is significantly higher in Timor-Leste compared to other countries in the same Asia Pacific Region such as Indonesia (12 %); the Philippines (11 %); Papua New Guinea (44 %); Malaysia (20 %); Australia (4 %); and Fiji (12 %) [13].

There are some possible reasons for the high prevalence of smoking among minors in Timor-Leste. Giving free cigarettes is part of tradition in some communities in Timor-Leste to express thanks, especially among people in lower socioeconomic status. Children under age are usually exposed to this tradition, which is an indication of smoking initiation among them. Another obvious reason is due to socioeconomic condition. Most underage children are forced to sell cigarettes on the side of the street to help sustain economic needs of the family. The money from selling cigarettes is used to buy books, pens and other family’s necessities. Another significant reason is due to inexistence of restrictive law to control cigarette sales and consumption by minors. Cigarettes are available in small shops and street vendors with retail price. Most cigarettes cost less than US$1 a pack. Ordinary people including children underage are able to buy even only 1 stick of cigarette from small shops and street vendors. Although every single pack of cigarette is labeled with health warnings, it is effectively meaningless to many smokers due to high illiteracy among poor people, especially in rural settings. Therefore, it is clear that the accessibility and affordability of cigarettes are very high in Timor-Leste.

### Influence of parental and peer smoking

Our analysis found that both parental and close friends smoking were independently associated with current smoking prevalence among minors (ages 13–15) in Timor-Leste. Having parents and close friends who smoke can be a significant factor to influence smoking initiation among minors. This finding is supported by some literatures conducted in other countries. For example, prevalence of young Latinos who smoked in the United States was found to be associated with having both parents and friends who were smokers at baseline [14]. The study concluded that having parents and peers smoking could have higher acceptability of smoking among minors and may influence them to start initiating cigarette smoking. Consequently, the association between parental and peer smoking and prevalence of smoking among minors can indicate easy availability and accessibility of cigarettes within home, which is obvious in the case of Timor-Leste.

### Environmental tobacco smoke or second-hand smoke

Our analysis revealed that although harmful effects of smoking was discussed by the family, the rates of current smokers among students in places such as schools, public places and homes were found to be statistical significantly associated. This suggests that exposure to environmental tobacco smoke (ETS) or second-hand smoke (SHS) especially at schools, public places and homes remains a big concern in Timor-Leste. Statistic from the 2006 GYTS showed that youth exposure to SHS at home in Timor-Leste was 63 %, which positioned Timor-Leste in the second highest after Indonesia (65 %) in the South-East Asia Region [13].

Moreover, some literatures have revealed that exposure to ETS can be associated with adverse health outcomes. Exposure to ETS at homes especially among children can prejudice them to be at high risk of getting diseases such as acute respiratory illnesses, asthma, ear infection and sudden infant death syndrome [15]. In Timor-Leste, 21 % of death among children under 5 are caused by acute respiratory infections and exposure to ETS is one of the risk factors [16]. Therefore, intervention such as health education or improving knowledge seems to help reduce children’s ETS exposure at homes. However, this strategy will be less effective to prevent smoking behavior and reduce ETS exposure without strong policy support such as restriction on smoking at home.

Indeed, mounting evidence suggests that legislation to ban smoking at homes has been found to reduce various health outcomes. For instance, ear problems and infant death syndrome related to ETS exposure in New South Wales, Australia were found to be reduced after implementing and enacting a legislation to ban smoking at homes [17]. In the case of Timor-Leste, comprehensive interventions through both family education and policy development to forbid smoking at public places and homes are required to reduce harm from SHS exposure especially among vulnerable populations. Protection of the environment from SHS to protect human health and wellbeing should be strategic, which must have a support from both leadership commitment and strategic policy settings.

### Tobacco related advertisements

A piece of literatures have revealed that public awareness and support through mass communication, health education and reliable information are essential elements for the success of tobacco control program [1, 4, 13]. However, our findings showed that current smoking status among minors in Timor-Leste was associated by factors such as billboard advertisements. The reason can be due to the fact that cigarette Brand Company such as LA from Indonesia has become one of the popular sponsors for social events such as sports and musical concerts. During these social events, cigarette products can be seen everywhere through billboard advertisements. This clearly shows that the cigarette company simultaneously advertises, markets and promotes all forms of tobacco. This tobacco marketing has been a substantial contributing factor to smoking among young people in the country, while restriction law is not in place.

Our finding in terms of the relationship between cigarette advertising and smoking rate among minors is consistent with studies in other developing country settings. For example, current smoking rate among young people in Indonesia has been found to be associated with exposure to tobacco related advertisements and anti-smoking campaigns [18]. In Indonesia, cigarette media campaigns have freely promoted their advertising in public places such as shopping malls as well as through mass media campaign on TV and radio [18].

### Policy implications

Public concern about the health effects of smoking has prompted a number of countries to adopt policies designed to reduce smoking rate among young people. Among these policies are: laws prohibiting sale of tobacco products to underage groups; restrictive advertising laws; and smoke-free areas regulation. In Timor-Leste, public awareness, education and support for tobacco control are unlikely to reduce smoking rates among young people without legislative intervention.

Timor-Leste must develop and enact a comprehensive policy to regulate and control the circulation of cigarettes across the country, particularly among underage groups. Developing a restriction law of the current tobacco sales to minors is extremely effective strategy to lower smoking prevalence and consumption among underage people [19]. This analysis recommends that Timor-Leste’s government should develop and enact a proper policy intervention that should include an article setting down a minimum age for those who smoke and sell cigarettes.

Moreover, schools in Timor-Leste have virtually no health education with regard to smoking. The whole school system has absolutely no regulation on tobacco use in school, whether among school kids or teachers. Evidence suggests that wider introduction of comprehensive school smoking policies helps reduce smoking among young people [20]. Thereby, school environment is critical to be target for tobacco control strategies and school tobacco policies should be considered as part of a comprehensive approach to tobacco use among minors [21]. This analysis recommends the government of Timor-Leste to implement a strategic intervention targeting school-based health education and to develop school tobacco policies. Therefore, both school education and tobacco policy interventions are essential to reduce smoking prevalence among in-school students in Timor-Leste.

Besides, since people in Timor-Leste have high exposure to SHS, it is necessary to forbid smoking in public places such as public transport, health facilities, offices and schools to reduce health outcomes associated with smoking as well as the smoking rate among population in the country. Timor-Leste should have a free-smoke policy to regulate and ban smoking in public places. This analysis suggests that smoking should be restricted in all government offices and schools as the starting point.

As of current smoking status among minors has been found to be highly associated with exposure to billboard advertisements, Timor-Leste must establish a policy on comprehensive bans on all forms of tobacco advertising, marketing, sponsorship and promotion. This policy approach has been found to be effective in reducing young people smoking rates, as observed in other low and middle-income country settings [13].

### Strengths and limitations

There are some strengths in our analysis. A major strength is dataset used is a nationally representative, which was specifically designed to collect tobacco survey among young people in Timor-Leste. Another strength is the study can be generalizable to the young population in the same age with current smoking status in Timor-Leste. Our analysis highlights the importance of developing national smoking policy to support education and behavioral change interventions. However, our research has some limitations. One limitation has been the type of study, which is a cross-sectional study (Additional file 1) that allows us to only analyze the association, but not the cause and effects (causality cannot be analyzed). Another limitation has been the potential of recall bias due to the data is self-reported.

## Conclusion

Undoubtedly, smoking behavior among adolescents in Timor-Leste is associated with some determinant factors such as availability of the product, easy access or lack of restrictive laws and other factors such as family and peer smoking. In Timor-Leste, as a result of early exposure, smoking has increased the severity and spread of tuberculosis and other respiratory conditions, which remain high among adolescents in the country [22]. Despite the known health problems associated with tobacco use through family health education and anti-smoking messages, young people in Timor-Leste continue to initiate and develop regular patterns of tobacco use.

Our findings reveal that current smoking among minors is higher among boys (59 % as compared to girls 28 %), and among the age of more than 15 (57 %). Some immediate actions are possible, such as developing age restriction and smoke free policies. Our analysis highlights the importance of developing national smoking policy to support education and behavioral change interventions that should be considered by policy makers in Timor-Leste. Findings from our analysis is essential to allow Timor-Leste’s government to develop the first national smoking policy to be existed in the country after 15 years of independence as well as to inform decision-making in order to allocate resources in the tobacco control sector. Therefore, this analysis provides evidence-based information for developing comprehensive tobacco control programs - both education and policy interventions to reduce smoking rate among young people in Timor-Leste.

## Additional file

Additional file 1:
**STROBE Statement—Checklist of items that should be included in reports of** ***cross-sectional studies.*** (PDF 127 kb)

## Abbreviations

CDC: center for disease prevention and control
CI: confidential intervals
ETS: environmental tobacco smoke
FCTC: framework convention on tobacco control
GYTS: global youth tobacco survey
IBM: international business machines
MoH: ministry of health
OR: odds ratio
SHS: second-hand smoke
SPSS: statistical package for the social sciences
WHO: World Health Organization
